# Editorial: Gastropoda immunity and host-pathogen interactions

**DOI:** 10.3389/fimmu.2024.1385106

**Published:** 2024-03-22

**Authors:** Joanna M. Bridger, Matty Knight

**Affiliations:** ^1^Life Sciences, Brunel University London, Uxbridge, United Kingdom; ^2^Department of Sciences and Mathematics, University of District of Colombia, Washington, ME, United States; ^3^School of Medicine and Health Sciences, George Washington University, Washington, DC, United States

**Keywords:** gastropoda disease, gastropoda immunity, *Biomphalaria*, *Pomacea*, *Marisa*

This Research Topic on gastropod innate defense is a timely and important contribution toward filling an existing information gap on a topic that covers a large taxonomic class within the phylum mollusca. Gastropods, commonly known as snails and slugs are numerous, diverse, and widespread in environments ranging from freshwater to dry land. Their survival throughout millennia is a testament to an ability to adapt and thrive by possessing an innate defense system that we still do not completely understand. The breath and scope of the manuscripts provided here in this Research Topic on immunity in gastropods reflects the growing interest in research in this group of invertebrates (Alba et al.; de la Ballina et al.; Friesen et al.). Accordingly, most of the reviews are about the freshwater snail, *Biomphalaria glabrata*, because it has been maintained for several years in a laboratory, and for its role in the transmission of *Schistosoma mansoni*, the causative agent of schistosomiasis in the Western Hemisphere (Lu et al.). This fact notwithstanding, the immunobiology of other species of snails, namely the apple snails, *Pomacea canaliculata*, and *Marisa cornuarietis*, were also reviewed (Rodriquez et al.). Interestingly, this was within the context of settling discrepancies in the biological processes that have been described previously for how humoral and cellular defences are generated to intruders in these snails. The narrative in this descriptive manuscript was interesting and informative but many might think of as controversial. At the heart of these reviews and original articles is the fact that we may know about the cellular and humoral components involved in gastropod immunity but lack information about biochemical and molecular mechanisms that orchestrate the protection of these organisms from the onslaught of foreign microbes and pathogens that confronts them regularly (Sheehy et al.). An excellent educational glossary on the plethora of infectious agents that can affect gastropods was also provided (O’Brien and Pellet).

Despite the ability to withstand infections, however, it is clear that certain pollutants might adversely challenge the survival of these organisms, especially if chemicals dampen the number of circulating hemocytes in changing environmental conditions (Lynch et al.).

As mentioned above, the snail, *B. glabrata*, transmitting its parasite, *S. mansoni*, is thus far the best studied in relation to the molecular interaction in this snail host-parasite interaction.

Reviews here on B. glabrata include the transcriptomics between infected and non-infected resistant and susceptible snails and results showing differences in gene expression between these two snails while responding to schistosomes in an environment that either rejects and kills the parasite, or where the parasite survives to complete the larval stage of its development (infectious cercarial stage) without recognition or destruction in a long incubation period that continues for the lifetime of the snail (Lu et al.). While molluscs have been utilized as good model organisms, especially in the neurosciences. It is clear from this editorial topic that gastropods are emerging as convenient organisms to study in a variety of research subjects.

For example, investigations about molecular determinants involved in immunobiology of gastropods are leading to discovery and characterization of immune defense proteins, such as thioester containing molecules (Marquez et al.). Despite these advances being made, including recent sequencing of the exotic *B.pfferiferi* species that is prevalent throughout Africa and transmits *Schistosoma pfeifferi* (1), it is apparent that interest in gastropods has to be sustained, especially with young investigators if the momentum in research and discovery can be realized for many more years to come.

In summary, this Research Topic on gastropoda immunity provides a timely Research Topic of interesting and complex reviews. Each is a stand-alone, and points to the impact that studies in these gastropods (traditionally understudied) in the immunobiology discipline has grown and is attracting new investigators at a rapid pace.

## Author contributions

JB: Writing – original draft, Writing – review & editing. MK: Writing – original draft, Writing – review & editing.
